# CALENDAR

**DOI:** 10.1054/bjoc.2000.1448

**Published:** 2000-08-17


					27 September-22 November 2000
European School of Genetic Medicine:
Autumn 2000 courses
La Nunziata Centre, via Portobello, Sestri Levante, Genova,
Italy
27 September-1 October 2000 5th Course in Cancer Genetics and
Pediatric Oncology
13 October-16 October 2000 2 Courso di Consulenza Genetica
(in Italiano)
15 October-20 October 2000 2nd Course in Genetic Counselling
22 October-26 October 2000 1st Course in Bioinformatics
15 November-18 November 2000 1st Course in Genetics and
Renal Disease
19 November-22 November 2000 2nd Course in Molecular
Cytogenetics
Further information from: E-mail: eurgef@tin.it; Website:
www.eurogene.org
20-21 November 2000
191st Meeting of the Society for Endocrinology
Endocrine Related Cancer Symposium: EGFR superfamily
of receptors
Royal College of Physicians, London, UK
Further information from: Tel: + 44 (0) 1454 619 347; Fax: + 44
(0) 1454 616 671;
Website: www.endocrinology.org/SFE/confs.htm
11-13 December 2000
Genes & Cancer 2000
UK Molecular Biology & Cancer Network Meeting XVII
University of Warwick, UK
Further information from:
Transcriptional Control, Cytoplasmic Signalling, Nuclear
Structures, Cell Cycle Control, Therapeutics
Dr Stefan Roberts, Department of Biochemistry, University of
Dundee, Dundee, UK. Tel: + 44 (0) 1382 344248;
Fax: + 44 (0) 1382 345783; E-mail: s.g.e.roberts@dundee.ac.uk;
Website: www.icr.uk/ukmbcn/info.htm
5-7 February 2001
3rd UICC Cancer Management Meeting - Quality
Cancer Care in the New Millennium
Republic of Singapore
Further information from:
Secretariat, The National Cancer Centre (Singapore), 11 Hospital
Drive Level 4, Singapore 169610, Republic of Singapore.
Tel: + 65 436 8294/8283/8186; Fax: + 65 225 7559; E-mail:
3rduice@nccs.com.sg; Website: www.nccs.com.sg
14-17 February 2001
International Conference on Dietary Factors: Cancer
Causes & Prevention
Vienna, Austria
The following main areas will be covered:
1. Chemical analytical data on hazardous and protective
constituents of foods
2. Mutagens and antimutagens in the human diet
3. Post initiation effects of dietary factors
4. Human biomonitoring and risk assessment
5. Epidemiology, trends in nutrition and dietary recommendations
Further information from:
Johanna B. Baukovits, Institute for Cancer Research,
Borschkegasse 8a, A-1090 Vienna, Austria. Fax: + 43 142 77 96
51; E-mail: walter.paukovits@univie.ac.at
7-10 March 2001
4th International Symposium on Leukemia and
Lymphoma - molecular pharmacology and new
treatment modalities
Amsterdam, The Netherlands
Chairman: G.J.L. Kaspers, R. Pieters, P. Sonneveld and A.J.P.
Veerman
Abstract deadline: 1 December 2000
Further information from:
VU Conference Service, De Boelelaan 1105, NL-1081 HV
Amsterdam, The Netherlands. Tel: + 31 (0) 20 444 5790;
Fax: + 31 (0) 20 444 5825; E-mail: vu_conference@dienst.vu.nl
21-23 May 2001
UK Radiological Congress 2001 (UKRC 2001)
(Incorporating IOS & MED X RAY(R)
)
Wembley Conference & Exhibition Centre, London, UK
Further information from:
UKRC Secretariat, PO Box 2895, London W1A 5RS, UK. Tel: +
44 (0) 20 7307 1410/20; Fax: + 44 (0) 20 7307 1414; E-mail:
ukrc@dial.pipex.com; Website: www.ukrc.org.uk
CALENDAR
840
British Journal of Cancer (2000) 83(6), 840
(c) 2000 Cancer Research Campaign
doi: 10.1054/ bjoc.2000.1448, available online at http://www.idealibrary.com on

				

